# Spanish version of the Talent Development Environment Questionnaire for sport: Cultural adaptation and initial validation

**DOI:** 10.1371/journal.pone.0177721

**Published:** 2017-06-05

**Authors:** Javier Brazo-Sayavera, Pedro R. Olivares, Georgios Andronikos, Russell J. J. Martindale

**Affiliations:** 1 Sport Sciences Faculty, University of Extremadura, Caceres, Spain; 2 Instituto Superior de Educación Física, Universidad de la República, Rivera, Uruguay; 3 Instituto de Actividad Física y Salud, Universidad Autónoma de Chile, Talca, Chile; 4 School of Applied Sciences, Edinburgh Napier University, Edinburgh, United Kingdom; University of North Carolina at Chapel Hill, UNITED STATES

## Abstract

This study aimed to translate the Talent Development Environment Questionnaire into Spanish and provide an initial validation. A recommended methodology for translation and cultural adaptation of questionnaires was applied. Once this had been completed, three hundred and thirty-two young athletes completed the Talent Development Environment Questionnaire. The results revealed that the five factor solution Talent Development Environment Questionnaire was confirmed. With the exclusion of one item due to low factor loading, the Talent Development Environment Questionnaire-5 had robust statistical support for its factor structure (χ2 (df = 305) = 499.64, p<0.01, CFI = 0.90, RMSEA = 0.045, SRMR = 0.055). It also demonstrated adequate convergent and discriminant validity. While the internal reliability was lower than in previous studies, it revealed acceptable levels. Specifically the overall 27 item Talent Development Environment Questionnaire-5 had a Cronbach α score of .877, and the reliability scores for individual factors 1–5 were .622; .761; .658; .605; .602 respectively. As such, it is recommended that the Spanish Talent Development Environment Questionnaire-5 can be used with confidence in Spain in both applied and research settings.

## Introduction

### The importance of the Talent Development Environment

The identification and development of talented individuals in sport has become increasingly important over recent years, as the standard of sporting performance has grown. Increasing financial interest and commitment, in addition to increased professionalism means greater pressure to maximise effectiveness of developing young people by National Governing Bodies, sport academies and clubs alike [1]. While much emphasis has been placed on “the process of recognizing current participants with the potential to become elite players” (i.e. talent identification) and “the discovery of potential performers who are currently not involved in the sport in question” (i.e. talent detection) [2], relatively less resources have been placed on understanding and maximising the talent development process or the ‘Talent Development Environment’ (TDE—[1]). Martindale and colleagues [1] defined the TDE as ‘all aspects of the coaching situation’, giving particular regard to the overarching goals and modus-operandi required for effective development. Other definitions and considerations have also been presented in the literature. For example, Gagné [3] highlighted three environmental considerations of a TDE 1) milieu; 2) individual; and 3) provisions. While Henriksen and colleagues [4] focussed on the dynamic nature of the TD system, including elements on a micro, macro and cultural level.

While there are some subtle differences in the definitions for TDEs, the main point here is that TDEs within sport seem to be relatively under researched. For example, work by researchers such as Bloom and Sosniak [5], Côté and colleagues [6] and Durand-Bush and Salmela [7] has developed a good understanding of the types of experiences and stages that an athlete may go through to reach elite status, however less is understood about the overarching aims and modus operandi associated with an effective ‘development phase’ to elite status [8].

It is clear that the development of talent relies on the interplay between a whole host of different factors such as natural abilities, intrapersonal characteristics, environmental features, learning opportunities, and chance [9]. However, the talent development environment (TDE) represents one of the more controllable factors associated with success, including factors related to coaching, competition, coherence between significant others, relationships and long term planning [8, 10].

The fact that TDEs can be influenced by those designing or working within them, makes it especially important that effective and safe practice is understood and applied. However, the extent to which evidence based practice drives action within the sporting community is unclear. For example, to take a well-known example, early specialisation still appears to be common practice in many youth sports. While it is acknowledged in the literature that there are different routes to achieving elite status (e.g., [11–13]) and sports that require early peak performance such as gymnastics, may demand early specialisation [14], or for example in football, early engagement [15], there is evidence that for the majority of sports it is not necessary for the achievement of elite status [16, 17]. In fact, research has shown that early specialisation can be problematic, increasing the likelihood of burnout, dropout or injury. There is also evidence pointing to clear benefits of diversification and sampling at early stages in many sports, acting as a foundation not only for continued motivation and mental health, but also contributing to the level and sustainability of performance at elite adult stages [18]. However, the key argument here is not whether early specialisation is better or worse than early diversification, but the extent to which practice is underpinned by a critical appraisal of the evidence or simply driven by tradition and habit.

Research that has examined elite sport success has shown that broad features such as training facilities, coach education, financial commitment and talent identification and development (TID) systems impact on the level of elite sport success (e.g., [19]). As such, the importance of understanding the key features of such TID systems is clear, this is perhaps exemplified through the increasing interest in understanding factors that underpin effective development in youth sport (e.g., [11]) and sport academies (e.g., [20–22]).

### The development of a TDE assessment tool

In response to the challenge of facilitating evidence based practice within a youth sport and talent development context, Martindale and colleagues [23] developed and validated the Talent Development Environment Questionnaire (TDEQ). The TDEQ has been shown to be a practical and reliable tool that measures key holistic and generic processes involved in the effective long-term development of ‘talented’ athletes [23]. Specifically it is a seven factor, 59 item scale measuring the athlete experience within their respective talent environment, including 1) Long term development focus; 2) Quality preparation; 3) Communication; 4) Understanding the athlete; 5) Support network; 6) Challenging and supportive environment; and 7) Long term fundamentals. Work by Wang and colleagues [24] and Li and colleagues [25] focussed on refining the TDEQ due to a number of psychometric and practical concerns. For example, the long term development subscale contained a large number of items, and there was some conceptual overlap between factors [25]. This resulted in a 36 item, 6 factor TDEQ being used [24] and then subsequently a 25 item, 5 factor TDEQ (TDEQ-5) being developed through exploratory and confirmatory factor analysis. The TDEQ-5 represents a shortened (arguably more athlete friendly), and statistically robust questionnaire measuring 1) Long term development; 2) Holistic quality preparation; 3) Support network; 4) Communication; and 5) Alignment of expectations. Although it is important to note, that some concerns have been raised with regards to the ecological validity of shorter versions of the TDEQ, such as the 25 item TDEQ-5 in particular, where the pursuit of improved psychometric properties has been at the cost of potentially theoretically important items. However, it is recognised that the balance between conceptual and statistical quality is required and in line with this, Li and colleagues [25] presented the 36, 28 and 25 item TDEQ-5 for further investigation.

### The application of the TDEQ

Since the development of the TDEQ [23], a number of researchers have used it to examine different environments. This has included work that provides descriptive accounts of sport academy processes, and exploration of the relationship between TDEs and player outcomes such as progression, motivation, stress and well-being. For example, Martindale and colleagues [26] found that ‘quality preparation’ and ‘understanding the athlete’ were key discriminators of the effectiveness of sport academies in terms of player progression to senior elite status. Work by Wang and colleagues [24] found that the intrinsic nature of athlete goals was related to the nature of the environment. Specifically, long-term development focus and good support network positively predicted intrinsic goal striving.

Furthermore, Ivarsson and colleagues’ [27] study of 447 Swedish youth academy football players found that higher quality development environments (those which espoused a more long term and individualised coaching approach, with more established support networks) predicted higher psychological well-being and lower stress in youth players. Interestingly, they acknowledged, that even though evidence suggests that taking a long term development focus improves sought after outcomes (e.g., performance progression, elite status achievement, motivation and well-being), many are still influenced more by the pressure to ‘win’ games through the age group levels. This short-termism and lack of coherence between ‘significant others’ such as policy makers, coaches and parents has been highlighted previously as a barrier to successful talent development [28].

### The need for a Spanish TDEQ

In Spain (or any Spanish speaking countries), as far as the authors are aware, there are no specific questionnaires, which assess the influence of the environment on talent development processes. As such, it is important to develop an instrument designed to assess key environmental features for use within a Spanish context to allow international studies and cross-cultural comparisons, and carry out further work which examines the psychometric properties of the TDEQ. Given that work has already been carried out to develop and validate the TDEQ, it was considered appropriate and most efficient to adapt an internationally well-known, valid and reliable instrument like the TDEQ.

The aims of this study were twofold: first to translate into Spanish and adapt the TDEQ culturally following an international standardized methodology; and second, to examine the factor structure and psychometric properties of the TDEQ-5.

## Materials and methods

### Participants

Three hundred and twenty-two young Spanish regional level athletes (156 boys and 166 girls) from track and field (150), basketball (56), volleyball (51), Judo (33) and handball (32) took part in the study. The athletes were aged between 12 to 18 years (M = 14.6, SD = 1.3). They have trained in their sports between 1 and 5 years. All the participants were considered to be talented athletes by the nature of their status within the development pathway. All were currently selected members of regional talent programs and representative squads, and had been selected to take part in their respective national championships.

### Translation and cultural adaptation process

A recommended methodology for the translation and cultural adaptation of questionnaires was applied, which included forward and backward translation and cognitive interviews [29, 30].

Phase 1: Forward translation. First, two native Spanish translators with knowledge about the area of research conducted two forward translation versions of the TDEQ: each translator independently produced a forward translation of the original items, instructions and response options. To produce a combined version, both translators discussed the two translations and agreed on a single version with the aim to produce a conceptually and semantic equivalent translation of the original questionnaire, which was easy to understand [31]. This process permitted additional changes to the original version where words or concepts were untranslatable, or where words or terms had a specific meaning in one language but a semantically different or secondary meaning in the Spanish language.

Phase 2: Backward translation. The next step was to backward translate the combined Spanish version of the TDEQ into English by a native speaker of English who was fluent in Spanish. The backward translated of the questionnaire was then compared to the original version by the author of the original English TDEQ to detect any misunderstandings or inaccuracies in the translation process.

Phase 3: Cognitive interviews. The final phase was to administer the translated questionnaire to a sample of respondents to determine whether the translation (items, instructions and responses options) was acceptable, whether it was easy to understand and to evaluate the instrument’s clarity. This was tested by means of cognitive interviews using ‘probing’ and ‘paraphrasing’ methodology to provide respondents feedback in respect to errors or misunderstandings produced by the translation process [29, 30]. Such cognitive interview techniques are known to minimize measurement error introduced by the translation process and enable respondent misunderstandings to be rectified [32].

Cognitive interviews were face to face and were conducted in an egalitarian manner by a native Spanish speaker with 10 additional participants aged 15 to 20 years old.

The interviews consisted of:

1. An evaluation of the ease of comprehension of each item using dichotomous response options of either: 1) clear and comprehensible or 2) difficult to understand.
2. An evaluation of the ease of comprehension of each item using a numerical rating scale from 0 to 10 (0 very easy to understand to 10 very difficult to understand).
3. An investigation of individuals’ interpretations of questionnaire items with suggestions for improvements by asking those interviewed to express in their own words the perceived meaning of each item and then to re-phrase each item to verify their understanding.

### Procedure

The participants were mainly minors. They were taking part in training camps or competitions without parents' presence. So, it was impossible to get the written document. Coaches and caretakers had a written consent from their parents to decide to take part in different activities during the training camp or competition. The project was explained to the responsible persons and then, when they gave their consent it was explained to the participants. They were informed that it was not mandatory and they could not answer if they did not want. All of them took part in the study without problem. The potential participants and coaches were informed in detail about the experimental procedures and the possible risks and benefits of the project, which was approved by the Bioethics Committee of the University of Extremadura (Ref: 95/2015), and carried out according to the Declaration of Helsinki. The translated questionnaires were filled during the days leading into the respective national championships, where athletes took part. Administration of the questionnaires took place in quiet classroom conditions under the supervision of a researcher and was completed between 15 and 30 min. Participants were informed that there were no right or wrong answers, given assurance about the confidentiality of their responses, and encouraged to be honest and to ask questions if necessary.

### Data analysis

The factor structure of the TDEQ-5 [25] was examined with confirmatory factor analysis (CFA) using IBM SPSS Amos. The observed data was examined to test the goodness of fit for hypothesized model. Several commonly recommended indices were used to assess goodness of fit. These included comparative fit index (CFI), the standardized root mean square residual (SRMR), and the root mean square error of approximation (RMSEA) [33]. Goodness of fit is considered adequate if the CFI score is .90 or above, with an SRMR value under 0.08 and an RMSEA value under .60 [34]. Internal consistency was examined for the best-fit model using Cronbach α coefficients for the factors and total scale, where anything above .60 is considered adequate and above .70 good [35]. Discriminant validity was also examined and was considered robust if the confidence interval of estimated correlations between the latent factors does not exceed 1.00 [36]. Factor loading estimates provide an indication of the item level of convergent validity was also tested, where factor loadings higher than .50 and ideally greater than .707 were considered satisfactory [37, 38]. Means, standard deviations, high and low scores were calculated for the factors of the TDEQ-5.

## Results

### Translation and cultural adaptation process

Following a joint discussion between the translators about some of the words, concepts and terms used, and analysing the data of the cognitive interviews, the comprehensibility of all items included in the Spanish TDEQ-5 was considered good.

### Psychometric properties of Spanish TDEQ

Before the CFA was carried out, missing values in the data set were examined with the Little’s MCAR test to assess whether missing values were missing at random, before data imputation was considered [39]. The Little’s MCAR test revealed that missing values were missing at random (chi square = 506.659, df = 606, p = .061), subsequently, missing values were imputed using expectation-maximisation technique to replace the missing values. Table 1 presents goodness-of-fit statistics for the TDEQ-5. The RMSEA and SRMR values were found to be adequate, however the CFI was below the accepted .90 value. As such, the 28 item TDEQ-5 model did not show good fit: TDEQ-5 (5 factor) χ2 (df = 340; N = 322) = 603.99, p<0.01, CFI = .87, RMSEA = .049, SRMR = .057), and as such, was subsequently an additional CFA was conducted on all items with factor loadings above .60 [40]. This resulted in the exclusion of one item (item 15). The modified TDEQ-5 consisted of 5 factors and 27 items. Specifically, Factor 1 –Long Term Development Focus (items 19, 20, 22, 23, 25, 28); Factor 2 –Holistic Quality Preparation (items 2, 5, 10, 11, 12, 13, 17); Factor 3 –Support Network (items 1, 9, 7, 18, 26, 27); Factor 4 –Communication (items 6, 4, 8, 21) and; Factor 5 –Alignment of Expectations (items 3, 14, 16, 24). This modified Spanish TDEQ-5 showed adequate fit: Spanish TDEQ-5 χ2 (df = 305; N = 322) = 499.64, p<0.01, CFI = .90, RMSEA = .045, SRMR = .055).

**Table 1:** Goodness-of-fit statistics for TDEQ-5 & modified TDEQ-5.

Models	*df*	*χ2*	P	RMSEA	CFI	SRMR	AIC	BIC	ECVI
***TDEQ-5 CFA*** 5 Factor TDEQ-5 (28-item)	340	603.99	.00	.049	.87	.057	735.99	985.11	2.29
***Modified TDEQ-5 CFA*** 5 Factor Spanish TDEQ-5 (27-item)	305	499.64	.00	.045	.90	.055	645.64	921.19	2.01

The internal reliability of the TDEQ was deemed adequate given all values were above .60. Specifically, the Cronbach α score for the total scale was .877, and the Cronbach α scores for the individual factors 1–5 were .622; .761; .658; .605; .602 respectively. Discriminant validity of the scale was also supported as the latent factor correlations ranged from .33 to .639, with none of the 95% confidence interval correlation coefficients exceeding 1.00. Factor loading estimates provided an indication of a satisfactory level of convergent validity, with all factor loadings higher than the recommended .50. Means and standard deviations can be seen in Table 2.

**Table 2:** Internal consistency and descriptive statistics of the Spanish TDEQ-5 (n = 322).

	Cronbach α	Mean (SD)	High:Low
Total Scale	.877	2.49 (.66)	1.04:4.67
Factor 1 (Long Term Development Focus)	.622	2.17 (.67)	1.00:4.50
Factor 2 (Holistic Quality Preparation)	.761	2.62 (.97)	1.00:5.29
Factor 3 (Support Network)	.658	2.62 (.84)	1.00:5.17
Factor 4 (Communication)	.602	2.36 (.86)	1.00:5.50
Factor 5 (Alignment of Expectations)	.617	2.67 (.92)	1.00:5.75

## Discussion

The need and usefulness for evidence-based practice to drive the development of sporting talent is clear. Currently, there is one measurement tool that attempts to facilitate the application of theory to practice within TDEs. However, while this tool (TDEQ) has been translated into different languages for use internationally, and also developed further psychometrically, as yet there has been no Spanish version developed or validated. Given the number of Spanish speaking countries that have strong interest in effective talent development within sport, this was considered to be an important step.

The results of cognitive interviews showed that comprehensibility of all items included in the Spanish TDEQ-5 was good. After the exclusion of one item (item 15) due to low factor loading (< .60), the Spanish TDEQ-5 showed adequate factor fit through CFA. The final Spanish TDEQ-5 included five factors, specifically, Factor 1 –Long Term Development Focus (items 19, 20, 22, 23, 25, 28); Factor 2 –Holistic Quality Preparation (items 2, 5, 10, 11, 12, 13, 17); Factor 3 –Support Network (items 1, 9, 7, 18, 26, 27); Factor 4 –Communication (items 6, 4, 8, 21) and; Factor 5 –Alignment of Expectations (items 3, 14, 16, 24). The examination of the psychometric properties of the resulting model revealed adequate convergent validity and divergent validity. While the internal reliability was slightly lower than reported for the English version of the TDEQ-5 (.79 to .86 –Li et al., 2015), all factors revealed internal reliability above the minimum recommendations of .60, ranging from .602–.761, more similar to the range reported for the internal reliability of the revised TDEQ (.62-.85 –[24]). It is difficult to speculate, but it is possible that the translation process has led to some slight meaning loss or misinterpretation across items, or given the participants were slightly younger than in previous studies, there may be more likelihood of errors relating to misinterpretation of items or guessing questions [41].

Given the complexity inherent in TDEs, and therefore the challenge of ensuring strong construct validity, there is some concern about the loss of an item, in an already relatively short questionnaire. The item that was removed in order to improve the model fit was item 15 “I am involved in most decisions about my sport development”, is related to the ‘Alignment of Expectations’ factor, which captures the extent to which goals for sport development are coherently set and aligned (e.g., goal setting, goal review, and individualised). This item is the only item that directly considers an athletes involvement in decisions about their development. This fact, with the strong theoretical support for encouraging athlete input for motivational purposes (e.g., [42, 43]), is potentially problematic. As such, its exclusion could impact on the ecological validity of the TDEQ-5. As such, the decision to eliminate this item on statistical reasons needs to be taken with caution and further research is recommended to examine this further. Due to the need to strike a sensible balance between statistical examination, and the possibility of error within factor analysis [44] and the strength of the research evidence, the full 28 item TDEQ-5 has been presented in the S1 Table to allow future research to investigate this further. Indeed, it is clear that the nature of the environment associated with talent development is a complex and integrated situation, which adds complexity to the ability to ‘delineate between factors’ effectively [23].

Given the adequate psychometric properties of the Spanish TDEQ-5, it is recommended that this can be used with confidence in both applied and research settings. More specifically, it is recommended that the Spanish TDEQ-5 be used for monitoring, formative assessment and on-going TDE development and refinement, rather than summative evaluation of TDE quality. In a research context, the Spanish TDEQ-5 can be used to increase our understanding of the importance and relevance of different features of TDEs, as well as how the key factors relate to different outcomes. The tool can also be used to monitor and/or guide interventions.

Cognitive interviews were done to participants aged 15 and older, however the Spanish TDEQ was applied to participants aged 12 and older. Given the cognitive development between 12 and 15 we cannot guarantee the correct understanding of all items. However, during the measurement process we asked participants for any question they did not understand and participants under 15 did not report any problem. Additionally, due to the complexity of TDEs more work on the applicability of the TDEQ across different contexts and cultures is welcome, as is the continued examination of its psychometric properties, for example, test retest reliability, concurrent, criterion or ecological validity.

## Conclusions

This work provides evidence of the successful translation, development and validation of the Spanish TDEQ-5, which can be used with confidence in Spanish speaking research and applied contexts.

## Supporting information

S1 Table Spanish TDEQ-5 –factors and items. (DOCX)
