# Supplementary material for: Spanish version of the Talent Development Environment Questionnaire for sport: Cultural adaptation and initial validation
Source: PLoS One. 2017 Jun 5;12(6):e0177721. doi: 10.1371/journal.pone.0177721 (PMC5459334; doi:10.1371/journal.pone.0177721)
Supplement: S1 Table — (DOCX) [file pone.0177721.s001.docx]

Spanish TDEQ-5 – Factors and Items

| **Scale**  Spanish TDEQ-5 | **Items (Spanish)**  Likert scale 1 – 6 (Strongly agree; Agree; Agree a little bit; Disagree a little bit; Disgaree; Strongly disagree)  * reverse items | **Items (English)**  Likert scale 1 – 6 (Strongly agree; Agree; Agree a little bit; Disagree a little bit; Disgaree; Strongly disagree)  * reverse items |
| --- | --- | --- |
| **Factor 1: Long-term development**  The extent to which developmental programmes are specifically designed to facilitate athletes’ long-term success (e.g., fundamental training and rounded development, ongoing opportunities, and de-emphasis of winning). | 19. Mi entrenamiento está específicamente diseñado para ayudar a desarrollarme eficazmente a largo plazo  20. Empleo la mayor parte de mi tiempo desarrollando habilidades y cualidades que mi entrenador me dice que necesitaré si voy a competir con éxito a máximo nivel o nivel profesional  22. Mi entrenador/a me permite aprender a través de mis propios errores  23. Me darían buenas oportunidades incluso si experimentara un descenso en el rendimiento  25. Mi entrenador/a le da más importancia a lo que hago en el entrenamiento y la competición que a ganar  28. Mi entrenador/a enfatiza la necesidad del trabajo constante de habilidades fundamentales y básicas | 19. My training is specifically designed to help me develop effectively in the long term.  20. I spend most of my time developing skills and attributes that my coach tells me I will need if I am to compete successfully at the top/professional level.  22. My coach allows me to learn through making my own mistakes.  23. I would be given good opportunities even if I experienced a dip in performance.  25. My coach emphasises that what I do in training and competition is far more important than winning.  28. My coach emphasizes the need for constant work on fundamental and basic skills. |
| **Factor 2: Holistic quality preparation**  The extent to which intervention programmes are prepared both inside and outside of sports settings (e.g., caring coach, clear guidance, mental preparation, and balanced life). | *2. Pocas veces me incitan a planear cómo actuar cuando las cosas pudieran ir mal  *5. Mi entrenador/a no parece estar interesado en mi vida fuera del deporte  *10. En mi deporte, las recomendaciones sobre lo que necesito para progresar no están muy claras  *11. No recibo mucha ayuda para desarrollar de forma efectiva mi fortaleza mental en el deporte  *12. Mi entrenador raramente utiliza tiempo para hablar con otros entrenadores que trabajan conmigo  *13. Mi entrenador raramente habla conmigo sobre mi bienestar  *17. No me han enseñado mucho sobre cómo equilibrar entrenamiento, competición y recuperación | *2. I am rarely encouraged to plan for how I would deal with things that might go wrong.  *5. My coach doesn’t appear to be that interested in my life outside of sport.  *10. The guidelines in my sport regarding what I need to do to progress are not very clear.  *11. I don’t get much help to develop my mental toughness in sport effectively.  *12. My coach rarely takes the time to talk to other coaches who work with me.  *13. My coach rarely talks to me about my well-being.  *17. I am not taught that much about how to balance training, competing, and recovery. |
| **Factor 3: Support network**  The extent to which a coherent, approachable, and wide-ranging support network is available for the athlete in all areas (e.g., professionals, parents, coaches, and schools). | 1. Puedo reunirme con mi entrenador/a u otro miembro del equipo técnico cuando lo necesito (Por ejemplo, fisioterapeuta, psicólogo/a, preparador físico, nutricionista, etc)  7. Aquellos que me ayudan en mi deporte están de acuerdo en qué es lo mejor para mi (Por ejemplo. Entrenadores, fisioterapeutas, psicólogos/as deportivos, preparadores físicos, nutricionistas, etc)  9. Actualmente, tengo acceso a una variedad de diferentes tipos de profesionales para ayudarme en mi desarrollo deportivo (Por ejemplo. Fisioterapeuta, psicólogo/a deportivo, preparador físico, nutricionista, etc)  18. Mis entrenadores hablan regularmente con el resto de personas que me apoyan en mi deporte sobre qué es lo que estoy intentando conseguir (Por ejemplo. Fisioterapeuta, psicólogo/a deportivo, nutricionista, preparador físico, etc)  26. Mis programas de entrenamiento están desarrollados específicamente en relación con mis necesidades  27. Mis entrenadores se aseguran que mi colegio/instituto/universidad entiendan mi situación con entrenamientos/competiciones | 1. I can pop in to see my coach or other support staff whenever I need to (e.g., physiotherapist, psychologist, strength trainer, and nutritionist).  9. Currently, I have access to a variety of different types of professionals to help my sports development (e.g., physiotherapist, sport psychologist, strength trainer, and nutritionist).  7. Those who help me in my sport seem to be on the same wavelength as each other when it comes to what is best for me (e.g. coaches, physiotherapists, sport psychologists, strength trainers, and nutritionists).  18. My coaches talk regularly to the other people who support me in my sport about what I am trying to achieve (e.g., physiotherapist, sport psychologist, nutritionist, strength and conditioning coach).  26. My training programmes are developed specifically to my needs g/competitions.  27. My coaches ensure that my school/university/college understand about me and my training/competition. |
| **Factor 4: Communication**  The extent to which the coach communicates effectively with the athlete in both formal and informal settings (e.g., development path, rationale for training, and feedback). | 4. Mi entrenador/a y yo hablamos sobre qué hicieron los actuales y/o antiguos deportistas de nivel mundial para triunfar  6. Mi entrenador/a y yo hablamos frecuentemente sobre aquello que necesito hacer para progresar hacia el nivel más alto en mi deporte (Por ejemplo. Comportamientos en el entrenamiento, rendimiento en competiciones, aspectos físicos, mentales, técnicos y tácticos  8. Normalmente mi entrenador y yo intentamos identificar cuál será mi próxima gran prueba/competición antes de que ocurra  21. Mi entrenador/a explica cómo mi programa de entrenamiento y competición están unidos para ayudarme a mejorar | 4. My coach and I talk about what current and/or past world-class performers did to be successful.  6. My coach and I regularly talk about things I need to do to progress to the top level in my sport (e.g., training ethos, competition performances, physically, mentally, technically, and tactically).  8. My coach and I often try to identify what my next big test will be before it happens.  21. My coach explains how my training and competition programme work together to help me develop. |
| **Factor 5: Alignment of expectations**  The extent to which goals for sport development are coherently set and aligned (e.g., goal setting, goal review, and individualised goals). | 3. Los consejos que me dan mis padres coinciden con los consejos que me dan mis entrenadores  14. Regularmente fijo los objetivos con mi entrenador, los cuáles son específicos para mi desarrollo individual  15. Estoy involucrado en la mayoría de decisiones sobre mi desarrollo deportivo  16. Mis entrenadores sacan tiempo para hablar con mis padres sobre mi y aquello que estoy intentando conseguir  24. Mi progreso y mi rendimiento personal son revisados regularmente de forma individual | 3. The advice my parents give me fits well with the advice I get from my coaches.  14. I regularly set goals with my coach that are specific to my individual development.  15. I am involved in most decisions about my sport development.  16. My coaches make time to talk to my parents about me and what I am trying to achieve.  24. My progress and personal performance is reviewed regularly on an individual basis. |
